# Interleukin-18 as a drug repositioning opportunity for inflammatory bowel disease: A Mendelian randomization study

**DOI:** 10.1038/s41598-019-45747-2

**Published:** 2019-06-28

**Authors:** Lauren E. Mokry, Sirui Zhou, Cong Guo, Robert A. Scott, Luke Devey, Claudia Langenberg, Nick Wareham, Dawn Waterworth, Lon Cardon, Philippe Sanseau, George Davey Smith, J. Brent Richards

**Affiliations:** 1 Department of Epidemiology, Biostatistics and Occupational Health, McGill University, Montreal, Canada; 2 Centre for Clinical Epidemiology, Lady Davis Institute for Medical Research, Jewish General Hospital, Montreal, Canada; 3 Computational Biology, Functional Genomics, GlaxoSmithKline, Stevenage, United Kingdom; 4 Celgene, Cambridge, Massachusetts USA; 5 MRC Epidemiology Unit, University of Cambridge, Cambridge, United Kingdom; 6 Target Sciences, GlaxoSmithKline, Upper Merion, Pennsylvania USA; 7 MRC Integrative Epidemiology Unit, Bristol Medical School, Bristol, United Kingdom; 8 Department of Human Genetics, McGill University, Montreal, Canada; 9 Department of Medicine, McGill University, Montreal, Canada; 10 Department of Twin Research and Genetic Epidemiology, King’s College London, London, United Kingdom

**Keywords:** Drug discovery, Genetics, Inflammatory bowel disease, Epidemiology

## Abstract

Support from human genetics increases the probability of success in drug development. However, few examples exist of successful genomically-driven drug repositioning. Given that a Mendelian form of severe enterocolitis is due to up-regulation of the interleukin-18 (IL18) signaling pathway, and pharmacologic inhibition of IL18 has been shown to reverse this enterocolitis, we undertook a Mendelian randomization study to test the causal effect of elevated IL18 levels on inflammatory bowel disease susceptibility (IBD) in 12,882 cases and 21,770 controls. Mendelian randomization is an established method to assess the role of biomarkers in disease etiology in a manner that minimizes confounding and prevents reverse causation. Using three SNPs that explained almost 7% of the variance in IL18 level, we found that each genetically predicted standard deviation increase in IL18 was associated with an increase in IBD susceptibility (odds ratio = 1.22, 95% CI = 1.11–1.34, P-value = 6 × 10^−5^). This association was further validated in 25,042 IBD cases and 34,915 controls (odds ratio = 1.13, 95% CI = 1.05–1.20). Recently, an anti-IL18 monoclonal antibody, which decreased free IL18 levels, was found to be safe, yet ineffective in a phase II trial for type 2 diabetes. Taken together, these genomic findings implicated IBD as an alternative indication for anti-IL18 therapy, which should be tested in randomized controlled trials.

## Introduction

The majority of drug development programs fail to produce medicines that gain regulatory approval^1,2^. This is most often due to lack of efficacy, rather than poor safety profiles^2^, leading to the suggestion that drug target identification and validation may be more successful using human genetics, since such an approach can offer direct insight into the perturbation of putative targets in humans. Indeed, recent evidence demonstrates that medicines aimed at therapeutic targets supported with human genetic evidence are more likely to gain regulatory approval than therapies lacking such support^3,4^.

Inflammatory bowel disease (IBD), which includes Crohn’s disease (CD) and ulcerative colitis (UC), is an inflammatory condition of the gastrointestinal tract for which there are many associated genetic loci^5,6^, but few available medications supported by human genetics data. The etiology of IBD remains poorly understood and as consequence approximately one-fifth of patients are unable to achieve remission using current therapies^7^. Recent evidence suggests that the members of the interleukin (IL) 1 family (including IL1α, IL1β, IL18 and IL33), which are established regulators of gastrointestinal inflammation, could influence IBD risk^8^.

Natural experiments from human genetics in the form of Mendelian disease, where proteins in the interleukin pathways have been altered due to rare mutations, lend support to this hypothesis. First, gain of function mutations in *NLCR4* (an important regulator of epithelial inflammasomes) lead to severe and chronic elevations in IL18 and also to early onset enterocolitis^9,10^. This suggests that IL18 could be a mediator of NLCR4-associated early onset enterocolitis. Human experimental evidence further supports this hypothesis. Recently, a child carrying a gain-of-function *NLCR4* mutation, developed severe enterocolitis and extremely elevated IL18 levels. The child was treated with recombinant IL18 binding protein, which decreased free IL18 levels to undetectable levels, impaired IL18 signaling, and resolved the severe enterocolitis^11^. In individuals with non-Mendelian forms of IBD, fresh intestinal biopsy samples demonstrated higher expression of IL18 levels in intestinal epithelial cells relative to controls^12^. This is concordant with evidence from murine models which found that knocking out IL18 signaling was protective against colitis and mucosal damage^13^. Thus evidence from Mendelian genetics, cellular expression studies and murine models all provide evidence of a role for IL18 signaling in intestinal inflammation. However, it is not yet fully understood whether common genetic perturbations of IL18 signaling can lead to increased susceptibility to IBD, the most common form of inflammatory enterocolitis.

**Figure 1:**
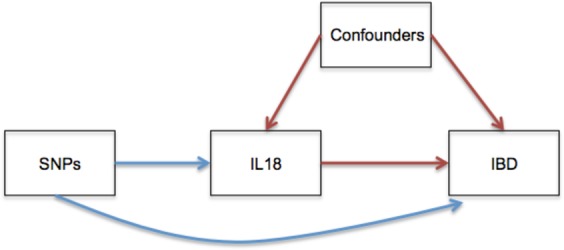
Directed Acyclic Graph. This figure displays the MR study design. Confounders are that are factors associated with both IL18 level and risk of IBD, yet do not lie in the causal pathway between IL18 and IBD. When confounding factors are unknown, or not adequately adjusted for in observational analyses, they bias estimates of the association between IL18 and IBD. In MR analyses, SNPs that influence IL18 level are tested for their effect on IBD instead of IL18 measurements. Since SNPs are randomized at conception, this breaks association with potentially confounding variables and allows for an estimate of the effect on IL18 on IBD risk.

We therefore tested whether variation in IL18 levels in the general population was causally associated with risk of IBD. To do so, we employed Mendelian randomization (MR) methods. MR has been used extensively to provide evidence to strengthen inference regarding causality of biomarkers, such as IL18, in disease etiology^14^. Under the assumptions of MR, if IL18 is causal in IBD pathology, then the genetic determinants of IL18 should influence disease risk (Fig. 1). A main advantage of the MR approach is that potential for confounding is greatly limited since allele assignment is randomized at conception, thus breaking potential association with confounders, much like the process of randomization in randomized controlled trials. Further, allele assignment always precedes disease onset, and is unaltered by it, thereby preventing reverse causation, where the disease itself influences the level of the exposure being studied.

If IL18 levels were causally related to an increased risk of IBD, this may have an immediate clinical impact since an IL18 inhibitor, GSK1070806, is already in development. GSK1070806 was developed for type 2 diabetes and recently tested in a phase II clinical trial which demonstrated a favourable safety profile, but failed to show any clinically relevant effects on glucose metabolism^15^. Evidence from MR may indicate whether this IL18 inhibitor could be repositioned to IBD.

## Methods

### Data sources

To investigate whether IL18 levels influence IBD susceptibility, we undertook a two-sample MR approach^16^ where summary statistics were selected from separate GWASs of IL18 levels^17^ and IBD risk^6^. An updated GWAS of IL18 levels^18^ and a recent larger IBD meta-analysis^19^ were also included for replication purposes. In addition, since disease severity may provide greater insight for drug target validation, we selected summary statistics from a recent GWAS of CD prognosis^20^. We selected summary statistics of SNPs that were genome-wide significant for IL18 levels (p < 1.2 × 10^−9^, which accounted for multiple testing cytokines) in recent a GWAS study of 41 cytokine traits involving up to 8,293 individuals of Finnish ancestry^17^. Corresponding effect estimates on IBD risk were obtained from the International IBD Genetics Consortium, which is the largest GWAS published to date for IBD (N = 12,882 cases, 21,770 controls)^6^. For the MR analysis of CD progression we selected summary statistics from a recently published CD prognosis GWAS using 1762 and 972 CD cases of poor and good prognosis respectively^20^. The methods of these GWAS are described in detail elsewhere^6,17,20^.

### Mendelian randomization analyses

MR has been used extensively to interrogate the role of modifiable risk factors in disease etiology^14,21,22^. In brief, the two-sample MR approach allows summary statistics to be used from separate GWAS for an exposure (such as IL18) and an outcome (such as IBD)^16^. This provides important advantages over traditional MR studies, where exposure and the outcome are measured in the same cohort, since summary statistics can be used from the largest genotyped cohorts of the exposure and outcome respectively. MR estimates for each SNP are then produced by the ratio method, where the SNP’s effect on IBD is weighted by its effect on IL18^16^. Individual MR estimates can then be combined using a fixed effects model to provide a summary measure^23,24^. To reduce the possibility of population stratification, we selected summary statistics from European ancestry-only analyses. We also performed a replication two-sample MR using exposure and outcome derived from different GWAS^18,19^. The summary statistics used for this analysis are publicly available and can be downloaded from their respective consortia’s website.

We performed sensitivity analyses to test the assumptions of MR and to assess for differential effects of IL18 across UC and CD. MR estimates were recalculated using the methods described above using summary statistics for only rs71478720, which lies in the IL18 gene and thus reduces the possibility pleiotropy biasing our results. Separate MR analyses were also performed for UC (N = 6,968 cases, 20,464 controls) and CD (N = 5,956 cases, 14,927 controls) respectively to assess for differential effects.

Since a monoclonal antibody for IL18 was previously tested in a phase II clinical trial for the treatment of T2D but failed to demonstrate efficacy, we sought to address whether MR analyses supported a role of IL18 in T2D risk. We used summary statistics from a large meta-analysis including samples from DIAGRAM, the UKBiobank and the EPIC-InterAct study (up to N = 26 903 T2D cases and 198 269 controls)^25^. We also performed a CD severity MR using summary statistics from the recently published GWAS for CD prognosis (N = 1762 poor prognosis and N = 972 CD good prognosis)^20^.

### Methods for evaluating the association of IBD at the IL18 receptor locus

There are additional lines of evidence implicating a role for IL18 in the etiology of IBD: SNPs within the IL18 receptor locus, harboring *IL18R1* (the Interleukin 18 Receptor 1) and *IL18RAP* (the IL18 receptor accessory protein) have also been associated with IBD^26–28^. To identify putative regulatory variants, we filtered variants in strong LD (R^2^ > 0.8) that resided within gene regulatory elements. Putative regulatory elements were defined by DNaseI hypersensitivity, H3K4me1, and H3K27ac in CD14, CD34, small intestine, and gastric tissues. The chromatin mark data was obtained from the ENCODE and Roadmap Epigenomics projects^29–31^. eQTL information for all associated variants was downloaded from the GTEx project^32^. All data was visualized using the UCSC Genome Browser^33,34^.

### Colocalisation Analyses

Biomarker MR studies may generate false positive associations when the causal SNP that influences the exposure is in linkage disequilibrium (LD) with a separate causal SNP that influences the outcome. It is therefore helpful to understand if there is a strong at separate signal at the locus which is in LD with the instrumental variable. Nevertheless, we undertook colocalization analysis using the coloc package^35^ and inspected the colocalisation plots to identify any potential confounding by LD.

### Phenome-Wide MR Study

To explore potential adverse effects of IL18 inhibition, we undertook MR analyses of all available outcomes on MR Base, an online repository and software package enabling simultaneous MR studies across multiple outcomes. We corrected for multiple testing using a Bonferroni correction of P < 0.05/3864^36^.

## Results

### Mendelian randomization analysis of IL18 levels on IBD susceptibility

Three SNPs were genome-wide significant (P < 1.2 × 10^−9^, accounting for multiple testing) for IL18 levels in the cytokine GWAS (N = 3,636): rs17229943 (*OCLN*), rs385076 (*NLRC4*) and rs71478720 (*IL18*) (Table 1)^17^. These three independent SNPs combined to explain 6.8% of the variation in IL18 levels^17^.

Two of the three variants have a mechanistic link to IL18 level. rs385076 is an intronic variant that maps to the *NLRC4 locus*. As described above, this gene product is an important regulator of inflammosomes and IL18 level^11,37^. rs71478720 lies in the intron of *IL18* and has been shown to influence IL18 expression in multiple tissues (including lung, pancreas, thyroid, skin and skeletal muscle) in the Genotype-Tissue Expression project (GTEx)^32,38^. rs17229943 is an intronic variant that lies near the occludin gene (*OCLN). OCLN* encodes an integral membrane protein important for the formation of tight junctions, but the mechanism in which it influences IL18 level remains unclear^39^.

Corresponding effect estimates of the same IL18 increasing alleles on risk of IBD were obtained from the International IBD Genetics Consortium, which is the largest GWAS published to date for IBD (N = 12,882 cases, 21,770 controls)^6^. Given this sample size and the fact that a relatively large proportion of variation in IL18 levels were attributed to these three SNPs, we had 100% statistical power to detect a 20% increase in odds of IBD per standard deviation increase in IL18^40^. All three IL18-increasing alleles were found to confer an increase in odds of IBD in the International IBD Genetics Consortium GWAS and IL18-increasing alleles at rs71478720 (*IL18)* and rs385076 (*NLCR4)* were individually associated with an increased risk of IBD (Table 1)^17^.

**Figure 2:**
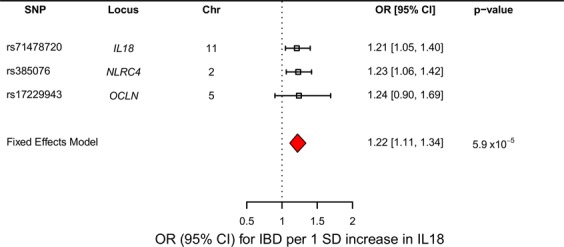
Forest Plot of the Results of the IL18-IBD MR Analysis. Forest plot of results where the boxes and confidence bars represent the individual MR estimates of the IL18 SNPs on IBD. The red diamond represents the summary estimate where all three SNPs were combined using a fixed effects model.

MR estimates were calculated by weighting each SNP’s effect on IBD by its effect on IL18. Each independent MR estimate demonstrated an effect of IL18 on risk of IBD (Fig. 2). When all three estimates were combined using a fixed effects model, we obtained a summary odds ratio of 1.22 (95% CI = 1.11–1.34, p-value = 5.9 × 10^−5^, *I*^2^ = 0%, 95% CI = 0–90%) per genetically predicted standard deviation increase in IL18. This evidence supports the hypothesis that increased IL18 levels increase risk of IBD.

### Assessment of Mendelian randomization assumptions

One important assumption of MR is that the chosen SNPs do not have effects on the outcome independent of the exposure (i.e. a lack of pleiotropy). Therefore to test for the presence of pleiotropy, we performed a sensitivity analysis where only rs71478720 (*IL18*) was used to calculate an MR estimate. Given that rs71478720 lies in an intron for *IL18* and influences expression of IL18 in multiple tissues^32,38^, this greatly reduces the possibility of pleiotropy since it is likely that this SNP acts directly through IL18 level. The MR estimate using this single SNP was similar to our meta-analysis (OR = 1.21, 95% CI = 1.05–1.40, p-value = 0.011), suggesting that our results were not substantially biased due to pleiotropy. Given that the cytokine signaling network is highly-correlated, we also queried whether the three IL18 SNPs associated with any of the other 40 cytokine traits tested in Ahola-Olli *et al*.^17^. Interestingly, only rs71478720 at *IL18*, was found be associated with interferon gamma-induced protein 10 (IP10) once corrected for multiple testing (p < 0.05/41 = 0.0012) (Supplementary Table 1). rs71478720 (*IL18*) exhibited a relatively small effect on ILP10 level (beta = 0.09, 95% CI = 0.037–0.14 p-value = 8.97 × 10^−4^) compared to its effect on IL18 l (beta = 0.27, 95% CI = 0.21–0.32, p-value = 3.71 × 10^−22^). Since rs71478720 acts as a cis-eQLT for IL18, it is likely that this SNP influences IL18 expression, which in turn modulates ILP10 level, given that IL18 itself has been shown to induce expression of interferon-gamma^41^.

### Replication MR analysis

Two genome-wide significant SNPs (surviving multiple corrections in both discovery and replication study) for IL18 levels in the cardiovascular disease biomarker GWAS were selected (N = 3,394): rs75649625 (IL18) and rs693918 (SRD5A2)^18^ (Supplementary Table 2). These two independent SNPs collectively explain 3.8% of the variation in IL18 levels. The estimates of IL18 increasing alleles for each SNP on risk of IBD were replicated using the latest IBD meta-analysis (N = 25,042 cases, 34,915 controls)^19^. Power calculation estimated that we had 99% statistical power to detect a 20% increase in odds of IBD per standard deviation increase in IL18.

Independent MR estimates of each SNP were calculated by weighting their effect on IBD by its effect on IL18 (Supplementary Fig. 1). We then combined the independent estimates using an inverse‐variance weighted model and obtained a summary odds ratio of 1.13 (95% CI = 1.05–1.20, p-value = 0.0021) per genetically predicted standard deviation increase in IL18, which replicated our initial MR findings.

### Separate Mendelian randomization analyses for CD and UC

We next tested whether this association with IL18 level was more pronounced in CD compared to UC. Meta-analyses investigating CD and UC as separate outcomes demonstrated a consistent effect of IL18 (OR_Crohn’s_ = 1.15, 95% CI = 1.01–1.31, p-value = 0.041; OR_UC_ = 1.25, 95% CI = 1.11–1.42, p-value = 2.39 × 10^−4^). This effect persisted for UC when only rs71478720 (*IL18*) was analyzed (OR = 1.26, 95% CI = 1.05–1.51, p-value = 0.012). For CD, the *IL18*-only analysis demonstrated the same direction of effect, however given the 95% confidence intervals, we cannot rule out no effect of IL18 on CD risk (OR = 1.10, 95% CI = 0.90–1.34, p-value = 0.37).

### Mendelian randomization analyses of IBD severity

These above MR findings reflect effects of IL18 on disease incidence and not disease progression since the GWAS for IBD employed a case-control design. As such, using summary statistics from an IBD susceptibility GWAS may not be the most appropriate method for drug target validation if interventions aim to treat the effects of the disease rather than prevention^42^. Since a relatively small GWAS (N = 2,734 CD cases) was recently published on CD prognosis^20^, we employed MR to interrogate the effect of IL18 on CD severity. We obtained inconclusive results (OR = 1.06, 95% CI = 0.77–1.46, p-value = 0.74, *I*^2^ = 0%, 95% CI = 0–90%) (Supplementary Fig. 2), which likely reflects low statistical power due to a relatively small sample size. In addition, progression MR analyses are prone to collider-bias, where the selection of cases can lead to a spurious inverse association with potential confounders^43^. This potential was demonstrated in the CD prognosis GWAS given that an inverse genetic correlation (as measured by LD score regression) was obtained for CD susceptibility and prognosis, although this correlation was estimated with wide 95% confidence intervals.

### The association of IBD and the IL18 receptor locus

The strongest association with IBD, rs917997 (P = 3.00 × 10^−20^, OR = 1.10)^26^, is found near the 5′ end of *IL18RAP* (Supplementary Fig. 3). The index SNP along with six other SNPs that are in near perfect LD reside within putative regulatory elements and promoters defined by open chromatin, active histone, and transcription factor binding sites in immune cell types (Supplementary Fig. 3, Supplementary Table 3). In blood cells, these SNPs are also eQTLs for IL18R1 and IL18RAP in the GTEx database (P = 1.4 × 10^−5^ and P = 7.3 × 10^−29^, respectively). These observations implicate IL18R1 and IL18RAP as effectors of the association at these loci and further support the involvement of the IL18 pathway in IBD.

### Mendelian randomization analyses of IL18 levels and type 2 diabetes susceptibility

Given that the previously published trial for IL18 inhibition in type 2 diabetes failed to demonstrate efficacy, we tested whether IL18 levels were causally associated with an increased risk of type 2 diabetes using the same MR methods described above. By using 26,903 individuals with type 2 diabetes and 198 269 controls, we found that MR analyses did not support a role of IL18 in type 2 diabetes risk (OR = 1.05, 95% CI = 0.99–1.13, P-value = 0.12) (Supplementary Fig. 4). Similar results were obtained in a previous MR study interrogating different but correlated SNPs (R^2^ > 0.05) mapping to *IL18* locus^44^. Therefore the MR of IL18 on type 2 diabetes is consistent with trial results and suggests that further investigation of this therapy for type 2 diabetes may not be successful. However, we note that again the GWAS for type 2 diabetes provided SNP-associations for susceptibility, rather than progression.

### Possible adverse effects of IL18 inhibition

To provide insights into potential side-effects of IL18 inhibition, we queried the three IL18 SNPs and proxies (R^2^ > 0.8) using PhenoScanner and MR base^36,45^, to understand if these SNPs were strongly associated with other traits that may produce adverse effects. Full results from PhenoScanner and MR base are provided in Supplementary Table 4 and Supplementary Dataset 1 respectively. Briefly our search on PhenoScanner demonstrated that none of the SNPs, or available proxies, were genome-wide significant for any trait other than IL18. rs385076 was nominally (p < 0.05/3 = 0.017) associated with depressive symptoms and overweight status, while rs71478720 was nominally associated with type 2 diabetes and fasting glucose. Information for rs17229943 was not available on PhenoScanner. In addition, we performed a hypothesis free MR of IL18 levels on all outcomes available on MR base. None of the results of these MR analyses were significant once we corrected for multiple testing (p < 0.05/3864 = 1.29 × 10^−5^). The results of these analyses are reported in Supplementary Dataset 1.

### Colocalisation

Inspection of colocalisation plots (Supplementary Fig. 5) showed no evidence of separate association signals for IL18 or IBD at the three instrumental variable loci which may have confounded our analyses.

### Phenome-Wide MR

Exploring for potential side-effects of IL18 inhibition we performed phenome-wide MR and identified no potential additional associations surviving Bonferroni correction.

## Discussion

Thus, our MR analyses support a role of IL18 in IBD susceptibility, with each genetically predicted standard deviation increase in IL18 conferring a 22% increase in odds of IBD (OR = 1.22, 95% CI = 1.11–1.34, p-value = 5.9 × 10^−5^). These findings may have an important clinical impact, given that anti-IL18 therapy (GSK1070806) was recently tested for the treatment of T2D, and considered safe, but ineffective. Further the results of our MR analysis of IL18 on T2D susceptibility were concordant with these trials results, providing additional evidence for the value of genetics to predict drug target success.

These findings have clinical relevance. First, epidemiological studies have indicated that the incidence of IBD is increasing across populations^46^. While there are many drugs indicated for the treatment of IBD, these drugs have varying degrees of efficacy to induce and maintain disease remission^7,47–49^. Targeting IL18 may provide benefit to IBD patients and our findings support investigating this question in clinical trials. However, while GSK1070806, was found to be well-tolerated among subjects with type 2 diabetes assigned to active treatment, the side effect profiles in IBD patients may be different. Further, given the small sample size of the phase II study and duration of therapy, the long-term adverse outcomes of GSK1070806 are not known. GSK1070806 is currently being tested in phase II clinical trials for renal transplantation^50^. This trial will better assess the tolerability and safety of GSK1070806 given the elevated risk of this patient.

This study has several limitations. Pleiotropy is a potential source of bias in MR studies and can be difficult to account for since the exact function of many genetic variants is unknown. However our MR estimate using only the *IL18* intronic variant is unlikely to be substantially influenced by pleiotropy since this has been shown to influence the expression of IL18 in multiple tissues. While this variant was nominally associated with IP10 in the cytokine GWAS, it is unclear whether this small effect upon IP10 level is independent of IL18. GTEx results indicate that rs71478720 (*IL18*) acts as a *cis*-eQTL for IL18 in multiple tissues, but importantly this did not include the colon, the most relevant tissue type for IBD. This may possibly be attributed to a small sample size (N = 124), or other technical issues such as cell-type variability and ascertainment. For instance there may be differences in *IL18* expression in intestinal epithelial versus smooth muscle cells not evaluated in GTEx. For our analysis, we cannot rule out possible bias due to residual European population stratification since summary estimates for IL18 were obtained from Finnish population whereas IBD estimates includes multiple European subgroups. Such bias would only occur if ancestry also associated IBD susceptibility. While the epidemiological evidence has demonstrated a north-west/south-east gradient for IBD, with the highest burden observed in the Nordic countries and the United Kingdom, it is unclear whether this is due to genetic factors or rather reflects variability in reporting and detection among the wealthier European nations^51^. Future GWAS of cytokine levels in other European populations can confirm our results. We also note that results of our CD progression MR were inconclusive. As data from large disease progression GWASs becomes increasingly available, this will enable MR to further validate effective drug targets.

Taken together, several lines of evidence strongly implicate a role for IL18 in IBD. These now include MR evidence demonstrating important effects of IL18 on IBD risk in the general population, Mendelian gain-of-function mutations leading to increased IL18 levels and severe enterocolitis, which was reversed by IL18 signaling inhibition, *cis-*eQTLs for IL18 receptors leading to increased IBD risk and finally intestinal biopsies from IBD patients showing increased IL18 expression in epithelial cells. Future clinical trials investigating anti-IL18 therapies for the treatment of IBD should help to test the utility of these approaches to identify clinically relevant drug targets for this important disease.

## Supplementary information

Supplementary Material

Dataset 1

## Data Availability

All data necessary to perform this MR analysis is included in Table 1 of the manuscript. Full summary statistics for the cytokine GWAS are available at: http://computationalmedicine.fi/data#Cytokine_GWAS. Dataset used for replication are available at: https://doi.org/10.5281/zenodo.264128. Full summary statistics for the IBD GWAS are available for download from the International IBD Genetics Consortium’s website at: https://www.ibdgenetics.org/downloads.html. Dataset used for replication are available at: ftp://ftp.sanger.ac.uk/pub/project/humgen/summary_statistics/human/2016-11-07/.

**Table 1:** Characteristics of SNPs used as instrumental variables.

Locus	SNP	Chr	IL18 increasing Allele	Allele Frequency	IL18 Results^a^		IBD Results^b^	
					Effect on IL18 (SD)	p-value	OR (95% CI)	p-value
*NLRC4*	rs385076	2	C	0.64	0.243	1.7 × 10^−22^	1.05 (1.02–1.09)	0.0051
*OCLN*	rs17229943	5	C	0.05	0.312	1.6 × 10^−11^	1.07 (0.97–1.18)	0.066
*IL18*	rs71478720	11	C	0.76	0.267	3.1 × 10^−22^	1.05 (1.01–1.09)	0.012

^a^effect size and p-value selected from the cytokine GWAS^17^.

^b^OR (95% CI) and p-value selected from the IBDGenetics Consortium^6^. Characteristics of SNPs used as instrumental variables. ^a^effect size and p-value selected from the cytokine GWAS^17^. ^b^OR (95% CI) and p-value selected from the IBDGenetics Consortium^6^.
