# Supplementary material for: Interleukin-18 as a drug repositioning opportunity for inflammatory bowel disease: A Mendelian randomization study
Source: Sci Rep. 2019 Jun 28;9:9386. doi: 10.1038/s41598-019-45747-2 (PMC6599045; doi:10.1038/s41598-019-45747-2)
Supplement: Supplementary file 1 — Supplementary Material [file 41598_2019_45747_MOESM1_ESM.docx]

**Supplementary Material**

TITLE: Interleukin-18 as a drug repositioning opportunity for inflammatory bowel disease: A Mendelian randomization study

**AUTHORS:** Lauren E Mokry,^1,2*^ Sirui Zhou,^1,2*^ Cong Guo,^3^ Robert A Scott,^3,5^ Luke Devey,^4^ Claudia Langenberg,^5^ Nick Wareham,^5^ Dawn Waterworth,^3^ Lon Cardon,^6^ Philippe Sanseau,^3^ George Davey Smith,^7^ J Brent Richards^1,2, 8, 9,10^

^1^ Department of Epidemiology, Biostatistics and Occupational Health, McGill University, Montreal, Quebec, Canada

^2^ Centre for Clinical Epidemiology, Department of Epidemiology, Lady Davis Institute for Medical Research, Jewish General Hospital, McGill University, Montreal, Quebec, Canada

^3^ Target Sciences, GlaxoSmithKline, Stevenage, United Kingdom

^4^ Celgene, Cambridge, Massachusetts, USA

^5^ MRC Epidemiology Unit, University of Cambridge, United Kingdom

^6^ Target Sciences, GlaxoSmithKline, Upper Merion, Pennsylvania, USA

^7^ MRC Integrative Epidemiology Unit, School of Social and Community Medicine, University of Bristol, Bristol, United Kingdom

^9^ Department of Human Genetics, McGill University, Montreal, Quebec, Canada

^9^ Department of Medicine, McGill University Montreal, Quebec, Canada

^10^ Department of Twin Research and Genetic Epidemiology, King's College London, United Kingdom

*These authors contributed equally to this study

**Supplementary Figure 1. IL18-IBD MR Validation Result**

**
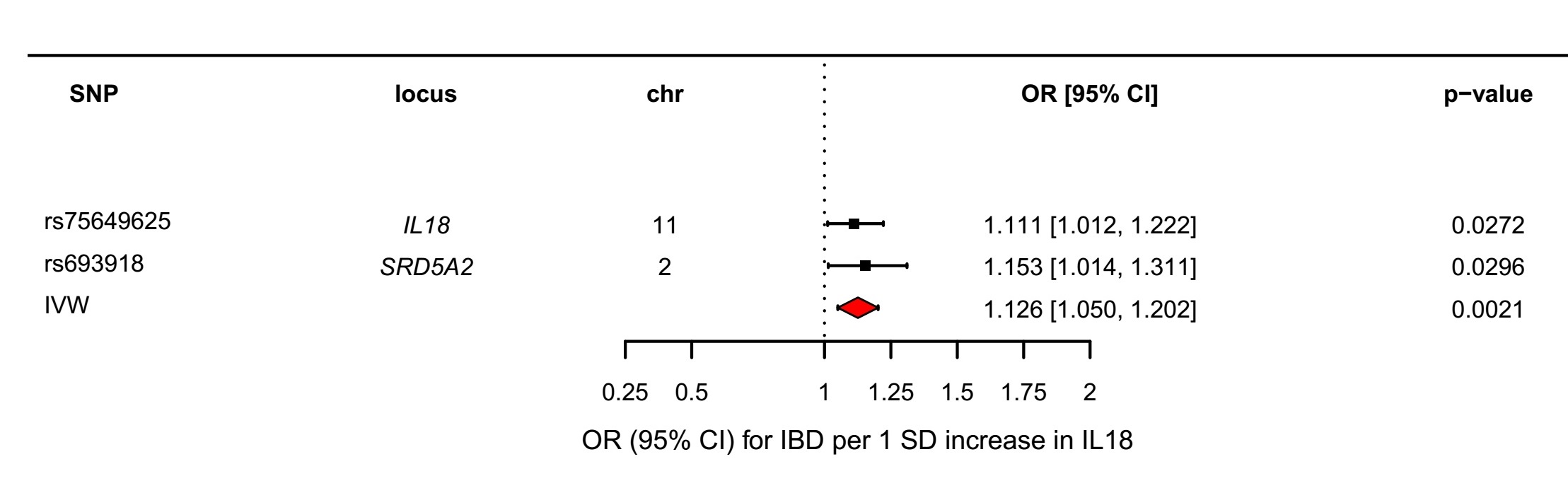
**

Forest plot of results where the boxes and confidence bars represent the individual MR estimates of the IL18 SNPs on IBD using the validation datasets in **Supplementary Table 2**. The red diamond represents the summary estimate where two SNPs were combined using fixed effects inverse‐variance weighted model.

**Supplementary Figure 2: IBD susceptibility versus severity forest plot**

This figure displays a forest plot of the results comparing the IBD susceptibility and severity MR analyses. The severity analysis only had 44% power to detect a 20% increase in odds of poor CD prognosis whereas the susceptibility analysis had 100% power to detect a 20% increase in odds of IBD. The small sample size in the CD prognosis GWAS contributed (n =1762 poor progression & 972 good progression CD cases) this decreased power to detect a causal relationship.

**Supplementary Figure 3: IBD association at the IL18 receptor locus.**

Peaks represent open chromatin and active enhancers in monocytes (CD14, CD19, MCD34), small intestine (SI), gastric tissue, B-cells (GM12878), and B-cells (Layered). IL18R1 and IL18RAP promoters are highlighted in blue. Putative regulatory variants are defined as SNPs in strong LD with the GWAS index SNP that also reside within a regulatory/open chromatin mark.

**Supplementary Figure 4: Forest Plot of the Results of the IL18-T2D MR Analysis**

Forest plot of results where the boxes and confidence bars represent the individual MR estimates of the IL18 SNPs on T2D. The red diamond represents the summary estimate where all three SNPs were combined using a fixed effects model. While in the IBD analysis SNPs at *IL18* were associated with increased risk of IBD, for T2D their estimates are centered around the null. Only SNPs at *OCLN* were associated with increased risk of T2D. The role *OCLN* on IL18 level is less defined and could be influenced by pleiotropy.

**Supplementary Figure 5: Colocalisation Analyses**

1. **Chromosome 2 locus: NLRC4**


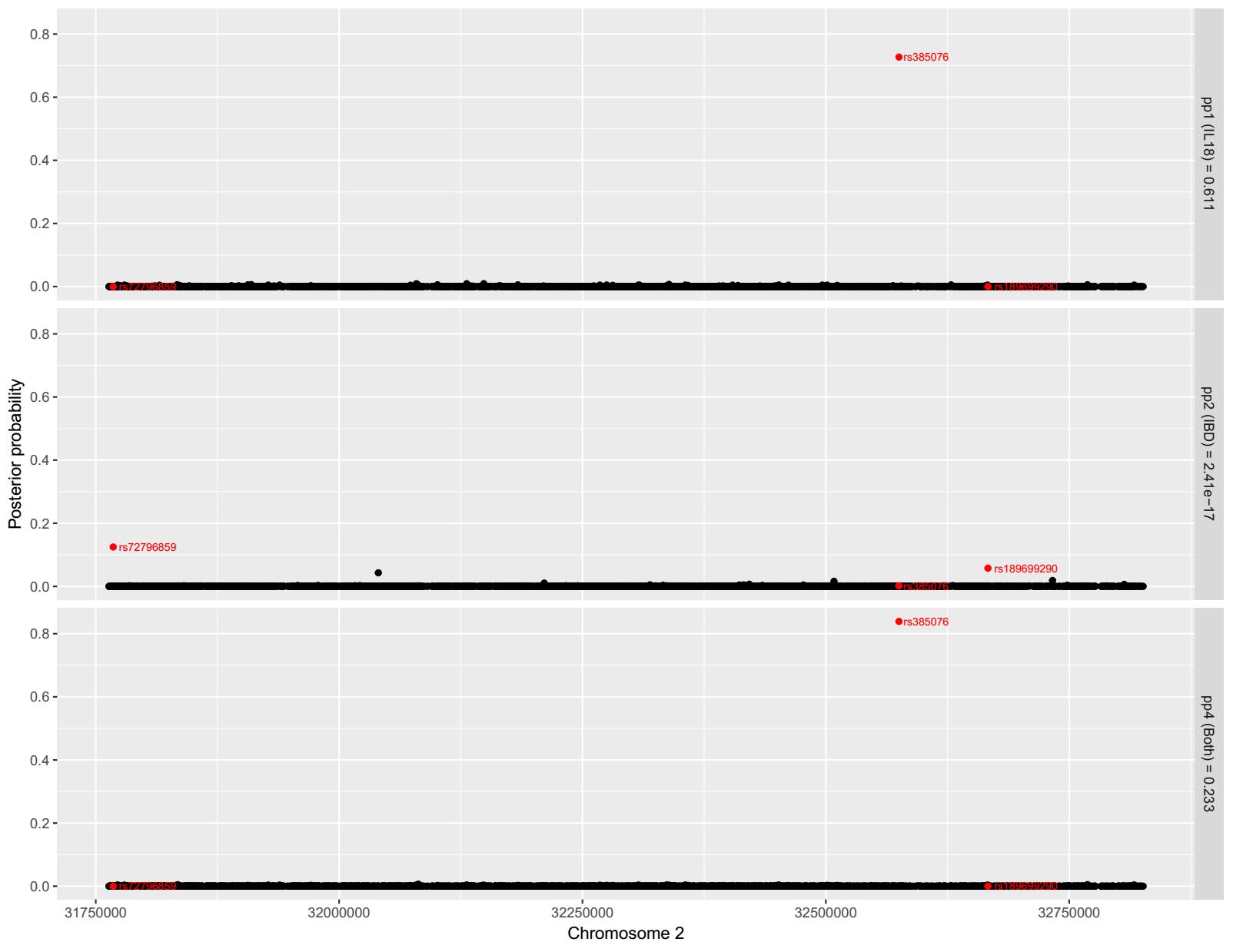


1. **Chromosome 5 Locus: OCLN**


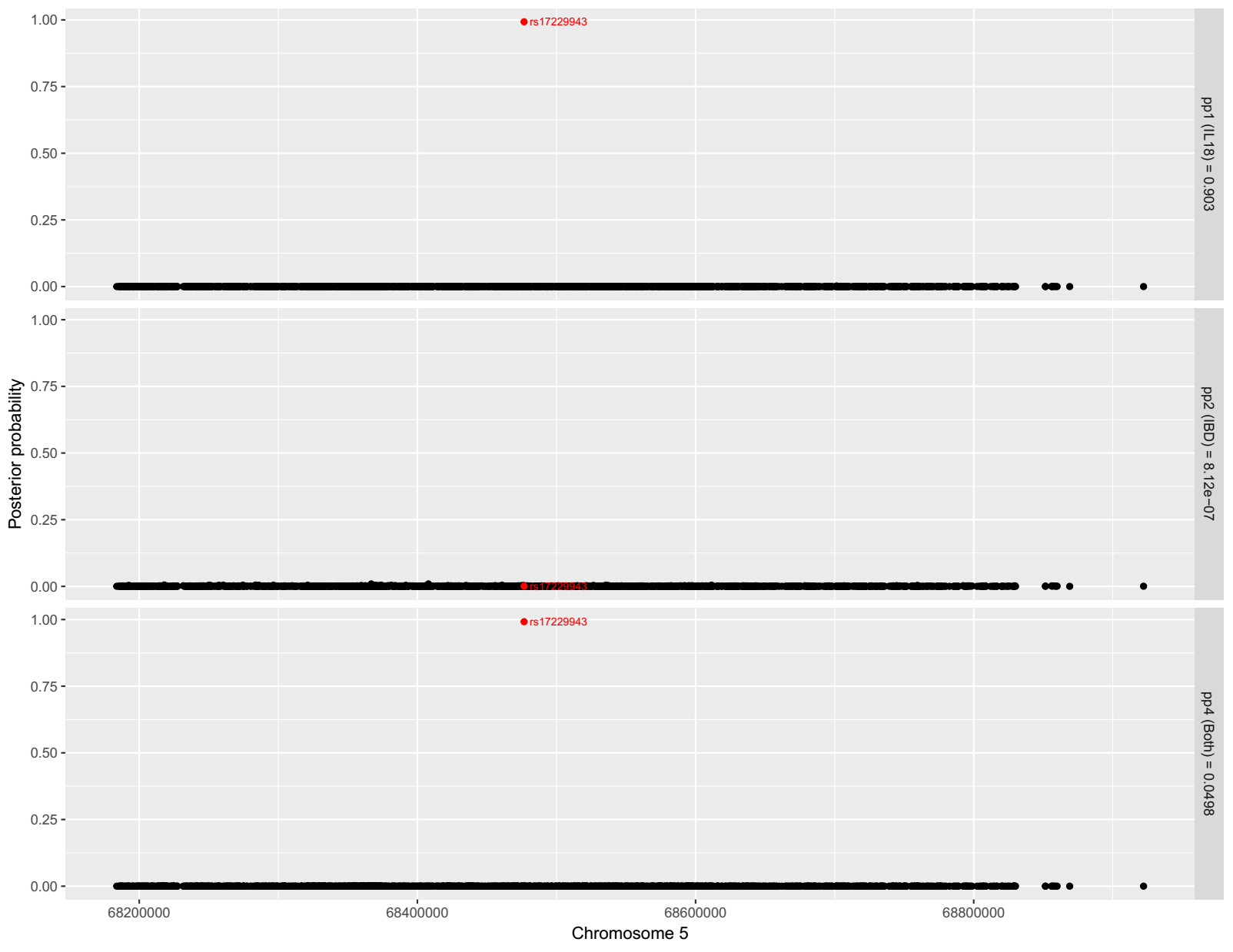


1. **Chromosome 11 Locus: IL18**


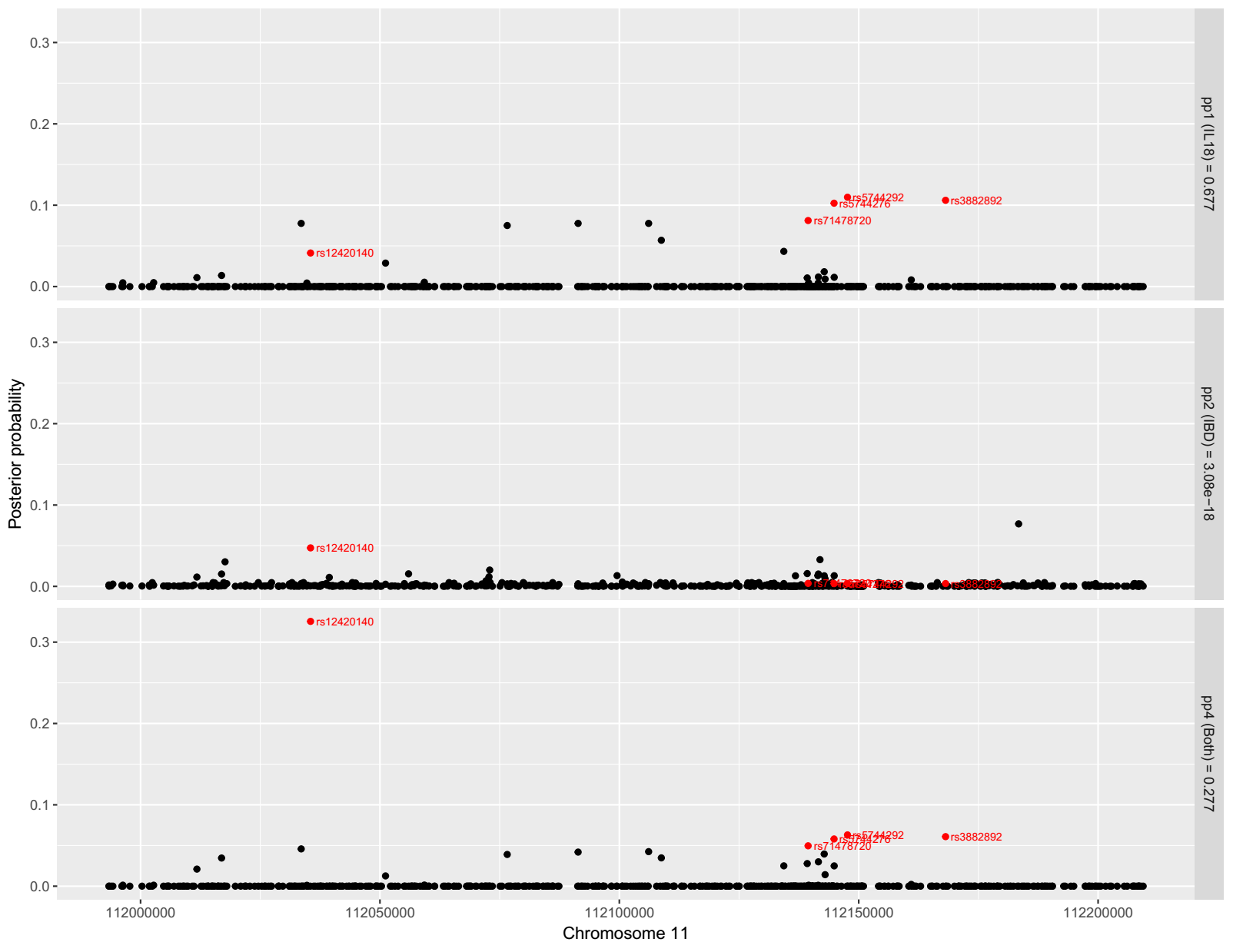


**Supplementary Table 1: Effect on IL18 SNPs on other cytokines**

| Cytokine | MarkerName | Chromosome | Position | OtherAllele | EffectAllele | Effect | StdErr | Direction | Pvalue | HetPVal |
| --- | --- | --- | --- | --- | --- | --- | --- | --- | --- | --- |
| bngf | rs385076 | 2 | 32489851 | t | c | -0.0052 | 0.0253 | +- | 0.8325 | 0.7351 |
| bngf | rs17229943 | 5 | 68682536 | a | c | 0.0388 | 0.047 | +- | 0.4095 | 0.2161 |
| bngf | rs71478720 | 11 | 112009605 | t | c | 0.0156 | 0.0279 | ++ | 0.577 | 0.9904 |
| ctack | rs385076 | 2 | 32489851 | t | c | 0.0143 | 0.0249 | ++ | 0.5678 | 0.8356 |
| ctack | rs17229943 | 5 | 68682536 | a | c | -0.0998 | 0.0461 | -- | 0.03 | 0.2401 |
| ctack | rs71478720 | 11 | 112009605 | t | c | -0.044 | 0.0275 | -- | 0.1157 | 0.1549 |
| eot | rs385076 | 2 | 32489851 | t | c | -0.0117 | 0.0167 | --+ | 0.4839 | 0.8405 |
| eot | rs17229943 | 5 | 68682536 | a | c | -0.0606 | 0.0317 | --+ | 0.05607 | 0.2226 |
| eot | rs71478720 | 11 | 112009605 | t | c | 0.0187 | 0.0185 | +-- | 0.3007 | 0.07296 |
| fgf | rs385076 | 2 | 32489851 | t | c | -4.00E-04 | 0.0172 | +-+ | 0.9916 | 0.6471 |
| fgf | rs17229943 | 5 | 68682536 | a | c | -0.0434 | 0.0324 | --- | 0.1762 | 0.7396 |
| fgf | rs71478720 | 11 | 112009605 | t | c | 0.0016 | 0.0193 | ++- | 0.9043 | 0.1266 |
| gcsf | rs385076 | 2 | 32489851 | t | c | -0.0092 | 0.0169 | --+ | 0.5948 | 0.08339 |
| gcsf | rs17229943 | 5 | 68682536 | a | c | -0.0246 | 0.032 | -++ | 0.4252 | 0.3031 |
| gcsf | rs71478720 | 11 | 112009605 | t | c | 0.037 | 0.0188 | ++- | 0.0463 | 0.1187 |
| groa | rs385076 | 2 | 32489851 | t | c | 0.0758 | 0.0252 | ++ | 0.002463 | 0.2753 |
| groa | rs17229943 | 5 | 68682536 | a | c | 0.0211 | 0.0472 | +- | 0.6528 | 0.392 |
| groa | rs71478720 | 11 | 112009605 | t | c | -0.0128 | 0.0279 | +- | 0.6547 | 0.5098 |
| hgf | rs385076 | 2 | 32489851 | t | c | 0.0042 | 0.0166 | --+ | 0.7877 | 0.2369 |
| hgf | rs17229943 | 5 | 68682536 | a | c | 0.0166 | 0.0314 | +++ | 0.6021 | 0.9395 |
| hgf | rs71478720 | 11 | 112009605 | t | c | 0.0369 | 0.0184 | +++ | 0.04558 | 0.9289 |
| ifng | rs385076 | 2 | 32489851 | t | c | 0.0038 | 0.0172 | -++ | 0.8245 | 0.8954 |
| ifng | rs17229943 | 5 | 68682536 | a | c | -0.0814 | 0.0324 | --+ | 0.01048 | 0.05427 |
| ifng | rs71478720 | 11 | 112009605 | t | c | 0.0381 | 0.0191 | +++ | 0.04582 | 0.7496 |
| il10 | rs385076 | 2 | 32489851 | t | c | -0.003 | 0.0172 | +-- | 0.8607 | 0.9642 |
| il10 | rs17229943 | 5 | 68682536 | a | c | -0.0399 | 0.0323 | -+- | 0.2104 | 0.1653 |
| il10 | rs71478720 | 11 | 112009605 | t | c | 0.0183 | 0.0191 | +++ | 0.337 | 0.9683 |
| il12p70 | rs385076 | 2 | 32489851 | t | c | -9.00E-04 | 0.0166 | +-+ | 0.9619 | 0.925 |
| il12p70 | rs17229943 | 5 | 68682536 | a | c | -0.0546 | 0.0316 | -+- | 0.0763 | 0.2057 |
| il12p70 | rs71478720 | 11 | 112009605 | t | c | 0.0177 | 0.0184 | ++- | 0.3291 | 0.2835 |
| il13 | rs385076 | 2 | 32489851 | t | c | -0.0095 | 0.0251 | -+ | 0.7099 | 0.6043 |
| il13 | rs17229943 | 5 | 68682536 | a | c | 0.0091 | 0.0466 | ++ | 0.8437 | 0.8686 |
| il13 | rs71478720 | 11 | 112009605 | t | c | 0.0129 | 0.0279 | ++ | 0.6383 | 0.712 |
| il16 | rs385076 | 2 | 32489851 | t | c | -0.0383 | 0.0253 | -- | 0.1298 | 0.921 |
| il16 | rs17229943 | 5 | 68682536 | a | c | -0.0045 | 0.0465 | -+ | 0.9222 | 0.5304 |
| il16 | rs71478720 | 11 | 112009605 | t | c | 0.0779 | 0.0281 | ++ | 0.005516 | 0.986 |
| il17 | rs385076 | 2 | 32489851 | t | c | -0.0108 | 0.0171 | --- | 0.5369 | 0.6896 |
| il17 | rs17229943 | 5 | 68682536 | a | c | -0.0536 | 0.0323 | --+ | 0.09433 | 0.5861 |
| il17 | rs71478720 | 11 | 112009605 | t | c | 0.0105 | 0.0191 | ++- | 0.5641 | 0.05215 |
| il18 | rs385076 | 2 | 32489851 | t | c | 0.2432 | 0.0248 | ++ | 1.66E-22 | 0.0707 |
| il18 | rs17229943 | 5 | 68682536 | a | c | 0.312 | 0.0463 | ++ | 1.62E-11 | 0.9826 |
| il18 | rs71478720 | 11 | 112009605 | t | c | 0.2669 | 0.0276 | ++ | 3.71E-22 | 0.586 |
| il1b | rs385076 | 2 | 32489851 | t | c | 0.0103 | 0.0197 | ++-+ | 0.2582 | 0.1584 |
| il1b | rs17229943 | 5 | 68682536 | a | c | -0.007 | 0.0366 | -+++ | 0.3035 | 0.8258 |
| il1b | rs71478720 | 11 | 112009605 | t | c | 0.0052 | 0.0219 | -++- | 0.5589 | 0.5537 |
| il1ra | rs385076 | 2 | 32489851 | t | c | -0.0165 | 0.0249 | -+ | 0.5236 | 0.251 |
| il1ra | rs17229943 | 5 | 68682536 | a | c | -0.019 | 0.0459 | -- | 0.6805 | 0.7624 |
| il1ra | rs71478720 | 11 | 112009605 | t | c | 0.0025 | 0.0275 | +- | 0.9079 | 0.1168 |
| il2 | rs385076 | 2 | 32489851 | t | c | -0.0126 | 0.0254 | -+ | 0.6404 | 0.1289 |
| il2 | rs17229943 | 5 | 68682536 | a | c | -0.0557 | 0.0473 | -- | 0.2391 | 0.8804 |
| il2 | rs71478720 | 11 | 112009605 | t | c | -0.0226 | 0.0281 | -- | 0.4306 | 0.3683 |
| il2ra | rs385076 | 2 | 32489851 | t | c | 0.0458 | 0.0247 | ++ | 0.06386 | 0.9424 |
| il2ra | rs17229943 | 5 | 68682536 | a | c | 0.0513 | 0.0457 | ++ | 0.2638 | 0.269 |
| il2ra | rs71478720 | 11 | 112009605 | t | c | 0.0394 | 0.0273 | ++ | 0.1469 | 0.609 |
| il4 | rs385076 | 2 | 32489851 | t | c | -0.0111 | 0.0167 | --- | 0.5062 | 0.9849 |
| il4 | rs17229943 | 5 | 68682536 | a | c | -0.0542 | 0.0319 | -++ | 0.08115 | 0.06882 |
| il4 | rs71478720 | 11 | 112009605 | t | c | 0.031 | 0.0187 | +-+ | 0.09618 | 0.6308 |
| il5 | rs385076 | 2 | 32489851 | t | c | -0.0161 | 0.0257 | -+ | 0.548 | 0.08115 |
| il5 | rs17229943 | 5 | 68682536 | a | c | 0.0857 | 0.0482 | ++ | 0.07901 | 0.05015 |
| il5 | rs71478720 | 11 | 112009605 | t | c | -0.007 | 0.0287 | +- | 0.8195 | 0.181 |
| il6 | rs385076 | 2 | 32489851 | t | c | -0.0025 | 0.0167 | +-- | 0.8876 | 0.7701 |
| il6 | rs17229943 | 5 | 68682536 | a | c | -0.0605 | 0.0315 | -++ | 0.04724 | 0.07238 |
| il6 | rs71478720 | 11 | 112009605 | t | c | 0.0328 | 0.0185 | +++ | 0.07481 | 0.6812 |
| il7 | rs385076 | 2 | 32489851 | t | c | -0.008 | 0.0256 | -+ | 0.7601 | 0.6348 |
| il7 | rs17229943 | 5 | 68682536 | a | c | 0.0074 | 0.0478 | -+ | 0.8831 | 0.6425 |
| il7 | rs71478720 | 11 | 112009605 | t | c | 0.0046 | 0.0285 | +- | 0.8682 | 0.8145 |
| il8 | rs385076 | 2 | 32489851 | t | c | -0.0183 | 0.0251 | -+ | 0.4827 | 0.07186 |
| il8 | rs17229943 | 5 | 68682536 | a | c | 0.0171 | 0.0467 | -+ | 0.7206 | 0.5248 |
| il8 | rs71478720 | 11 | 112009605 | t | c | 0.0238 | 0.028 | -+ | 0.4055 | 0.2478 |
| il9 | rs385076 | 2 | 32489851 | t | c | -0.0197 | 0.0248 | +- | 0.4174 | 0.2366 |
| il9 | rs17229943 | 5 | 68682536 | a | c | -0.0167 | 0.0462 | -+ | 0.7129 | 0.4362 |
| il9 | rs71478720 | 11 | 112009605 | t | c | 0.0118 | 0.0275 | +- | 0.6626 | 0.64 |
| ip10 | rs385076 | 2 | 32489851 | t | c | 0.0264 | 0.0247 | ++ | 0.2829 | 0.8957 |
| ip10 | rs17229943 | 5 | 68682536 | a | c | -0.0362 | 0.0457 | -- | 0.4279 | 0.6091 |
| ip10 | rs71478720 | 11 | 112009605 | t | c | 0.0907 | 0.0273 | ++ | 0.0008969 | 0.9307 |
| mcp1 | rs385076 | 2 | 32489851 | t | c | 3.00E-04 | 0.0166 | +-- | 0.9831 | 0.9293 |
| mcp1 | rs17229943 | 5 | 68682536 | a | c | -0.0097 | 0.0312 | -+- | 0.7513 | 0.5625 |
| mcp1 | rs71478720 | 11 | 112009605 | t | c | -0.0071 | 0.0184 | +-+ | 0.6863 | 0.09624 |
| mcp3 | rs385076 | 2 | 32489851 | t | c | 0.0231 | 0.0452 | -+ | 0.602 | 0.5583 |
| mcp3 | rs17229943 | 5 | 68682536 | a | c | 0.0439 | 0.0802 | ++ | 0.5995 | 0.687 |
| mcp3 | rs71478720 | 11 | 112009605 | t | c | -0.007 | 0.0492 | +- | 0.8958 | 0.697 |
| mcsf | rs385076 | 2 | 32489851 | t | c | 0.065 | 0.0302 | ++ | 0.03132 | 0.7497 |
| mcsf | rs17229943 | 5 | 68682536 | a | c | -0.0772 | 0.0544 | -+ | 0.1388 | 0.06582 |
| mcsf | rs71478720 | 11 | 112009605 | t | c | -0.01 | 0.0334 | -+ | 0.8227 | 0.01593 |
| mif | rs385076 | 2 | 32489851 | t | c | 0.0162 | 0.0253 | +- | 0.5259 | 0.4791 |
| mif | rs17229943 | 5 | 68682536 | a | c | 0.0535 | 0.0471 | ++ | 0.2507 | 0.3606 |
| mif | rs71478720 | 11 | 112009605 | t | c | -0.0267 | 0.028 | -- | 0.3451 | 0.6751 |
| mig | rs385076 | 2 | 32489851 | t | c | -0.0042 | 0.0246 | -+ | 0.873 | 0.4835 |
| mig | rs17229943 | 5 | 68682536 | a | c | 0.0167 | 0.0457 | -+ | 0.7115 | 0.09574 |
| mig | rs71478720 | 11 | 112009605 | t | c | 0.0616 | 0.0273 | ++ | 0.02353 | 0.6555 |
| mip1a | rs385076 | 2 | 32489851 | t | c | -0.0082 | 0.0252 | -+ | 0.7524 | 0.39 |
| mip1a | rs17229943 | 5 | 68682536 | a | c | -0.0393 | 0.0468 | -- | 0.399 | 0.4805 |
| mip1a | rs71478720 | 11 | 112009605 | t | c | -0.0191 | 0.0281 | +- | 0.5163 | 0.03606 |
| mip1b | rs385076 | 2 | 32489851 | t | c | -0.0181 | 0.0166 | +-- | 0.2777 | 0.3218 |
| mip1b | rs17229943 | 5 | 68682536 | a | c | -0.015 | 0.0313 | --+ | 0.612 | 0.4589 |
| mip1b | rs71478720 | 11 | 112009605 | t | c | 0.0076 | 0.0184 | ++- | 0.6707 | 0.547 |
| pdgfbb | rs385076 | 2 | 32489851 | t | c | 0.0114 | 0.0166 | +++ | 0.4929 | 0.8994 |
| pdgfbb | rs17229943 | 5 | 68682536 | a | c | -0.0571 | 0.0314 | --- | 0.06816 | 0.7206 |
| pdgfbb | rs71478720 | 11 | 112009605 | t | c | 0.0353 | 0.0184 | +-+ | 0.05616 | 0.4697 |
| rantes | rs385076 | 2 | 32489851 | t | c | -0.0329 | 0.0256 | -+ | 0.2115 | 0.04 |
| rantes | rs17229943 | 5 | 68682536 | a | c | -0.0635 | 0.0478 | +- | 0.182 | 0.01199 |
| rantes | rs71478720 | 11 | 112009605 | t | c | -0.0104 | 0.0285 | -+ | 0.6969 | 0.03304 |
| scf | rs385076 | 2 | 32489851 | t | c | 0.0063 | 0.0165 | +++ | 0.7036 | 0.9727 |
| scf | rs17229943 | 5 | 68682536 | a | c | 0.0048 | 0.0313 | --+ | 0.8972 | 0.3292 |
| scf | rs71478720 | 11 | 112009605 | t | c | -0.0057 | 0.0184 | -++ | 0.7483 | 0.1305 |
| scgfb | rs385076 | 2 | 32489851 | t | c | -0.0177 | 0.0246 | -- | 0.4755 | 0.7523 |
| scgfb | rs17229943 | 5 | 68682536 | a | c | -0.0144 | 0.0457 | +- | 0.7509 | 0.3195 |
| scgfb | rs71478720 | 11 | 112009605 | t | c | 0.0018 | 0.0273 | +- | 0.9392 | 0.5972 |
| sdf1a | rs385076 | 2 | 32489851 | t | c | -0.0129 | 0.0171 | +-- | 0.8524 | 0.6651 |
| sdf1a | rs17229943 | 5 | 68682536 | a | c | -0.037 | 0.0326 | -+- | 0.1561 | 0.6975 |
| sdf1a | rs71478720 | 11 | 112009605 | t | c | 0.0376 | 0.019 | +++ | 0.1993 | 0.7212 |
| tnfa | rs385076 | 2 | 32489851 | t | c | -0.0275 | 0.0254 | -- | 0.2799 | 0.8817 |
| tnfa | rs17229943 | 5 | 68682536 | a | c | 0.0321 | 0.0473 | ++ | 0.5024 | 0.5472 |
| tnfa | rs71478720 | 11 | 112009605 | t | c | -0.004 | 0.0283 | +- | 0.8915 | 0.6897 |
| tnfb | rs385076 | 2 | 32489851 | t | c | -0.0029 | 0.0382 | +- | 0.9028 | 0.07587 |
| tnfb | rs17229943 | 5 | 68682536 | a | c | -0.0387 | 0.0715 | +- | 0.5966 | 0.5532 |
| tnfb | rs71478720 | 11 | 112009605 | t | c | 0.0031 | 0.0414 | +- | 0.8925 | 0.07935 |
| trail | rs385076 | 2 | 32489851 | t | c | -0.0179 | 0.0166 | --+ | 0.2801 | 0.3501 |
| trail | rs17229943 | 5 | 68682536 | a | c | -0.0615 | 0.0316 | --- | 0.05247 | 0.907 |
| trail | rs71478720 | 11 | 112009605 | t | c | 0.0223 | 0.0185 | ++- | 0.2232 | 0.4847 |
| vegf | rs385076 | 2 | 32489851 | t | c | 0.0061 | 0.0179 | +-- | 0.7291 | 0.5658 |
| vegf | rs17229943 | 5 | 68682536 | a | c | -0.036 | 0.0336 | --- | 0.2832 | 0.8409 |
| vegf | rs71478720 | 11 | 112009605 | t | c | 0.0231 | 0.02 | ++- | 0.2422 | 0.498 |

**Supplementary Table 2: Characteristics of SNPs used as instrumental variables in validation study**

|  | | | | | **IL18 Results ^a^** | | **IBD Results ^b^** | |
| --- | --- | --- | --- | --- | --- | --- | --- | --- |
| **Locus** | **SNP** | **Chr** | **IL18 increasing Allele** | **Allele Frequency** | **Effect on IL18 (SD)** | **p-value** | **OR**  **(95% CI)** | **p-value** |
| *IL18* | rs75649625 | 11 | G | 0.76 | 0.289 | 1.46E-21 | 1.031 (1.003-1.058) | 0.027 |
| *SRD5A2* | rs693918 | 2 | G | 0.55 | 0.191 | 2.38E-11 | 1.028 (1.003-1.052 | 0.030 |

^a^ effect size and p-value selected from Folkersen et al, 2017

^b^ OR (95% CI) and p-value selected from de Lange et al, 2017

**Supplementary Table 3: SNPs that are in near perfect LD with rs917997 that are also eQTLs for IL18RAP and IL18R1**

| rsID | R^2^ with rs917997 | IL18RAP eQTL p-value (effect size) | IL18R1 eQTLp-value (effect size) |
| --- | --- | --- | --- |
| rs917997 | 1 | 7.2E-29 (0.31) | 1.40E-05 (0.11) |
| rs13015714 | 0.97 | 2.1E-29 (0.32) | 2.7E-05 (0.11) |
| rs1558627 | 0.97 | 2.1E-29 (0.32) | 1.2E-05 (0.11) |
| rs1420106 | 1 | 2.1E-29 (0.32) | 5.0E-06 (0.12) |
| rs6746271 | 1 | 6.7E-29 (0.31) | 1.4E-05 (0.11) |
| rs7559479 | 1 | 7.2E-29 (0.31) | 1.4E-05 (0.11) |
| rs1558650 | 1 | 7.2E-29 (0.31) | 1.4E-05 (0.11) |

**Supplementary Table 4: Results from Phenoscanner**

Part 1

| Locus | SNP | rsID | Pos (hg19) | Alleles | Proxy rsID | Proxy Pos (hg19) | Proxy Alleles | r2 | Dprime |
| --- | --- | --- | --- | --- | --- | --- | --- | --- | --- |
| *NLRC4* | rs385076 | rs385076 | chr2:32489851 | C/T | rs385076 | chr2:32489851 | C/T | 1 | 1 |
|  | rs385076 | rs385076 | chr2:32489851 | C/T | rs479333 | chr2:32489158 | G/C | 0.894 | 0.995 |
|  | rs385076 | rs385076 | chr2:32489851 | C/T | rs1265340 | chr2:32486917 | C/T | 0.894 | 0.995 |
|  | rs385076 | rs385076 | chr2:32489851 | C/T | rs62134033 | chr2:32479967 | G/A | 0.847 | 0.947 |
|  | rs385076 | rs385076 | chr2:32489851 | C/T | rs472101 | chr2:32478916 | T/G | 0.836 | 0.946 |
|  | rs385076 | rs385076 | chr2:32489851 | C/T | rs212718 | chr2:32461833 | A/C | 0.809 | 0.981 |
|  | rs385076 | rs385076 | chr2:32489851 | C/T | rs212739 | chr2:32404703 | G/A | 0.807 | 0.986 |
|  | rs385076 | rs385076 | chr2:32489851 | C/T | rs212739 | chr2:32404703 | G/A | 0.807 | 0.986 |
|  | rs385076 | rs385076 | chr2:32489851 | C/T | rs212717 | chr2:32461640 | A/G | 0.806 | 0.981 |
|  | rs385076 | rs385076 | chr2:32489851 | C/T | rs212715 | chr2:32460886 | G/C | 0.806 | 0.981 |
|  | rs385076 | rs385076 | chr2:32489851 | C/T | rs212745 | chr2:32412832 | G/C | 0.803 | 0.986 |
|  | rs385076 | rs385076 | chr2:32489851 | C/T | rs212760 | chr2:32429222 | G/A | 0.802 | 0.981 |
| *IL18* | rs71478720 | rs71478720 | chr11:112009605 | C/T | rs5744276 | chr11:112016871 | C/G | 1 | 1 |
|  | rs71478720 | rs71478720 | chr11:112009605 | C/T | rs5744276 | chr11:112016871 | C/G | 1 | 1 |
|  | rs71478720 | rs71478720 | chr11:112009605 | C/T | rs5744258 | chr11:112021767 | C/G | 0.994 | 1 |
|  | rs71478720 | rs71478720 | chr11:112009605 | C/T | rs5744256 | chr11:112022848 | A/G | 0.994 | 1 |
|  | rs71478720 | rs71478720 | chr11:112009605 | C/T | rs5744256 | chr11:112022848 | A/G | 0.994 | 1 |
|  | rs71478720 | rs71478720 | chr11:112009605 | C/T | rs1834481 | chr11:112023827 | C/G | 0.994 | 1 |
|  | rs71478720 | rs71478720 | chr11:112009605 | C/T | rs5744258 | chr11:112021767 | C/G | 0.994 | 1 |
|  | rs71478720 | rs71478720 | chr11:112009605 | C/T | rs5744256 | chr11:112022848 | A/G | 0.994 | 1 |
|  | rs71478720 | rs71478720 | chr11:112009605 | C/T | rs1834481 | chr11:112023827 | C/G | 0.994 | 1 |
|  | rs71478720 | rs71478720 | chr11:112009605 | C/T | rs1834481 | chr11:112023827 | C/G | 0.994 | 1 |
|  | rs71478720 | rs71478720 | chr11:112009605 | C/T | rs1834481 | chr11:112023827 | C/G | 0.994 | 1 |
|  | rs71478720 | rs71478720 | chr11:112009605 | C/T | rs5744222 | chr11:112037014 | G/T | 0.95 | 1 |
|  | rs71478720 | rs71478720 | chr11:112009605 | C/T | rs5744222 | chr11:112037014 | G/T | 0.95 | 1 |
|  | rs71478720 | rs71478720 | chr11:112009605 | C/T | rs7131094 | chr11:112044917 | C/T | 0.95 | 1 |
|  | rs71478720 | rs71478720 | chr11:112009605 | C/T | rs7131094 | chr11:112044917 | C/T | 0.95 | 1 |
|  | rs71478720 | rs71478720 | chr11:112009605 | C/T | rs12796114 | chr11:112049140 | A/C | 0.95 | 1 |
|  | rs71478720 | rs71478720 | chr11:112009605 | C/T | rs12797880 | chr11:112049191 | T/C | 0.95 | 1 |
|  | rs71478720 | rs71478720 | chr11:112009605 | C/T | rs12797880 | chr11:112049191 | T/C | 0.95 | 1 |
|  | rs71478720 | rs71478720 | chr11:112009605 | C/T | rs5744222 | chr11:112037014 | G/T | 0.95 | 1 |
|  | rs71478720 | rs71478720 | chr11:112009605 | C/T | rs5744222 | chr11:112037014 | G/T | 0.95 | 1 |
|  | rs71478720 | rs71478720 | chr11:112009605 | C/T | rs5744222 | chr11:112037014 | G/T | 0.95 | 1 |
|  | rs71478720 | rs71478720 | chr11:112009605 | C/T | rs7131094 | chr11:112044917 | C/T | 0.95 | 1 |
|  | rs71478720 | rs71478720 | chr11:112009605 | C/T | rs7131094 | chr11:112044917 | C/T | 0.95 | 1 |
|  | rs71478720 | rs71478720 | chr11:112009605 | C/T | rs7121554 | chr11:112048051 | C/T | 0.95 | 1 |
|  | rs71478720 | rs71478720 | chr11:112009605 | C/T | rs12796114 | chr11:112049140 | A/C | 0.95 | 1 |
|  | rs71478720 | rs71478720 | chr11:112009605 | C/T | rs12796114 | chr11:112049140 | A/C | 0.95 | 1 |
|  | rs71478720 | rs71478720 | chr11:112009605 | C/T | rs12797880 | chr11:112049191 | T/C | 0.95 | 1 |
|  | rs71478720 | rs71478720 | chr11:112009605 | C/T | rs12797880 | chr11:112049191 | T/C | 0.95 | 1 |
|  | rs71478720 | rs71478720 | chr11:112009605 | C/T | rs12420140 | chr11:112071294 | G/A | 0.816 | 0.964 |
|  | rs71478720 | rs71478720 | chr11:112009605 | C/T | rs12420140 | chr11:112071294 | G/A | 0.816 | 0.964 |
|  | rs71478720 | rs71478720 | chr11:112009605 | C/T | rs12420140 | chr11:112071294 | G/A | 0.816 | 0.964 |
|  | rs71478720 | rs71478720 | chr11:112009605 | C/T | rs12420140 | chr11:112071294 | G/A | 0.816 | 0.964 |
|  | rs71478720 | rs71478720 | chr11:112009605 | C/T | rs12420140 | chr11:112071294 | G/A | 0.816 | 0.964 |
|  | rs71478720 | rs71478720 | chr11:112009605 | C/T | rs12420140 | chr11:112071294 | G/A | 0.816 | 0.964 |
|  | rs71478720 | rs71478720 | chr11:112009605 | C/T | rs12420140 | chr11:112071294 | G/A | 0.816 | 0.964 |
|  | rs71478720 | rs71478720 | chr11:112009605 | C/T | rs12420140 | chr11:112071294 | G/A | 0.816 | 0.964 |
|  | rs71478720 | rs71478720 | chr11:112009605 | C/T | rs12420140 | chr11:112071294 | G/A | 0.816 | 0.964 |
|  | rs71478720 | rs71478720 | chr11:112009605 | C/T | rs12420140 | chr11:112071294 | G/A | 0.816 | 0.964 |
| *OCLN* | Index SNP or proxies not available on Phenoscanner | | | |  |  |  |  |  |

Part 2

| Locus | SNP | Proxy Indicator | Trait | Study | PMID | Year of Publication | Ancestry |
| --- | --- | --- | --- | --- | --- | --- | --- |
| *NLRC4* | rs385076 | 0 | Inflammatory bowel disease | IBDGC | 26192919 | 2015 | European |
|  | rs385076 | 1 | Ulcerative colitis | IBDGC | 23128233 | 2012 | European |
|  | rs385076 | 1 | Depressive symptoms | SSGAC | 27089181 | 2016 | European |
|  | rs385076 | 1 | Depressive symptoms | SSGAC | 27089181 | 2016 | European |
|  | rs385076 | 1 | Years of educational attainment in females | SSGAC | 27225129 | 2016 | European |
|  | rs385076 | 1 | Inflammatory bowel disease | IBDGC | 26192919 | 2015 | European |
|  | rs385076 | 1 | Overweight | GIANT | 23563607 | 2013 | European |
|  | rs385076 | 1 | Inflammatory bowel disease | IBDGC | 26192919 | 2015 | European |
|  | rs385076 | 1 | Overweight | GIANT | 23563607 | 2013 | European |
|  | rs385076 | 1 | Overweight | GIANT | 23563607 | 2013 | European |
|  | rs385076 | 1 | Overweight | GIANT | 23563607 | 2013 | European |
|  | rs385076 | 1 | Overweight | GIANT | 23563607 | 2013 | European |
| *IL18* | rs71478720 | 1 | Type II diabetes | DIAGRAM | 24509480 | 2014 | Mixed |
|  | rs71478720 | 1 | IL18 females | He M | 20150558 | 2010 | European |
|  | rs71478720 | 1 | Type II diabetes | DIAGRAM | 24509480 | 2014 | Mixed |
|  | rs71478720 | 1 | Type II diabetes | DIAGRAM | 24509480 | 2014 | Mixed |
|  | rs71478720 | 1 | Ulcerative colitis | IBDGC | 23128233 | 2012 | European |
|  | rs71478720 | 1 | Type II diabetes | DIAGRAM | 24509480 | 2014 | Mixed |
|  | rs71478720 | 1 | IL18 females | He M | 20150558 | 2010 | European |
|  | rs71478720 | 1 | IL18 females | He M | 20150558 | 2010 | European |
|  | rs71478720 | 1 | Interleukin 18 | He M | 20150558 | 2010 | European |
|  | rs71478720 | 1 | Interleukin 18 levels | He M | 20150558 | 2010 | European |
|  | rs71478720 | 1 | IL18 females | He M | 20150558 | 2010 | European |
|  | rs71478720 | 1 | Type II diabetes | DIAGRAM | 24509480 | 2014 | Mixed |
|  | rs71478720 | 1 | Systemic lupus erythematosus | Hom G | 18204098 | 2008 | European |
|  | rs71478720 | 1 | Type II diabetes | DIAGRAM | 24509480 | 2014 | Mixed |
|  | rs71478720 | 1 | 2 hour fasting glucose | MAGIC | 20081857 | 2010 | European |
|  | rs71478720 | 1 | 2 hour fasting glucose | MAGIC | 20081857 | 2010 | European |
|  | rs71478720 | 1 | Type II diabetes | DIAGRAM | 24509480 | 2014 | Mixed |
|  | rs71478720 | 1 | 2 hour fasting glucose | MAGIC | 20081857 | 2010 | European |
|  | rs71478720 | 1 | Differential exon level expression of PIH1D2 probe 3391225 in brain cortex | Heinzen | 19222302 | 2008 | European |
|  | rs71478720 | 1 | IL18 females | He M | 20150558 | 2010 | European |
|  | rs71478720 | 1 | IL18 | Melzer D | 18464913 | 2008 | European |
|  | rs71478720 | 1 | IL18 females | He M | 20150558 | 2010 | European |
|  | rs71478720 | 1 | 2 hour glucose | MAGIC | 20081857 | 2010 | European |
|  | rs71478720 | 1 | IL18 females | He M | 20150558 | 2010 | European |
|  | rs71478720 | 1 | IL18 females | He M | 20150558 | 2010 | European |
|  | rs71478720 | 1 | 2 hour glucose | MAGIC | 20081857 | 2010 | European |
|  | rs71478720 | 1 | IL18 females | He M | 20150558 | 2010 | European |
|  | rs71478720 | 1 | 2 hour glucose | MAGIC | 20081857 | 2010 | European |
|  | rs71478720 | 1 | Type II diabetes | DIAGRAM | 24509480 | 2014 | Mixed |
|  | rs71478720 | 1 | Height in males | GIANT | 23754948 | 2013 | European |
|  | rs71478720 | 1 | Systemic lupus erythematosus | Hom G | 18204098 | 2008 | European |
|  | rs71478720 | 1 | Inflammatory bowel disease | IBDGC | 26192919 | 2015 | European |
|  | rs71478720 | 1 | Ulcerative colitis | IBDGC | 23128233 | 2012 | European |
|  | rs71478720 | 1 | Ulcerative colitis | IBDGC | 26192919 | 2015 | European |
|  | rs71478720 | 1 | 2 hour fasting glucose | MAGIC | 20081857 | 2010 | European |
|  | rs71478720 | 1 | IL18 females | He M | 20150558 | 2010 | European |
|  | rs71478720 | 1 | 2 hour glucose | MAGIC | 20081857 | 2010 | European |
|  | rs71478720 | 1 | IL18 | Melzer D | 18464913 | 2008 | European |
| *OCLN* | Index SNP or proxies not available on Phenoscanner | | | | |  |  |

Part 3

| Locus | SNP | Source | Effect Allele | Association Alleles | EAF | MAF | Beta | SE | P | Direction | P Het |
| --- | --- | --- | --- | --- | --- | --- | --- | --- | --- | --- | --- |
| *NLRC4* | rs385076 | IBDGC | C | C/T | 0.636 | 0.364 | 0.04971 | 0.0177 | 0.005065 | + | 0.7906 |
|  | rs385076 | IBDGC | G | G/C | NA | NA | NA | NA | 0.00705 | NA | NA |
|  | rs385076 | SSGAC | C | C/T | 0.5951 | 0.4049 | -0.01 | 0.004 | 0.007229 | - | NA |
|  | rs385076 | SSGAC | G | G/A | 0.6063 | 0.3937 | -0.009 | 0.004 | 0.009838 | - | NA |
|  | rs385076 | SSGAC | T | T/G | 0.5037 | 0.4963 | 0.011 | 0.004 | 0.00261 | + | NA |
|  | rs385076 | IBDGC | A | A/C | 0.592 | 0.408 | 0.0442 | 0.0171 | 0.009965 | + | 0.5517 |
|  | rs385076 | GIANT | G | G/A | 0.75 | 0.25 | 0.025 | 0.0091 | 0.0049 | + | NA |
|  | rs385076 | IBDGC | G | G/A | 0.593 | 0.407 | 0.0439 | 0.017 | 0.009821 | + | 0.6344 |
|  | rs385076 | GIANT | A | A/G | 0.758 | 0.242 | 0.025 | 0.0093 | 0.0069 | + | NA |
|  | rs385076 | GIANT | G | G/C | 0.758 | 0.242 | 0.024 | 0.0093 | 0.0098 | + | NA |
|  | rs385076 | GIANT | G | G/C | 0.75 | 0.25 | 0.025 | 0.0091 | 0.0049 | + | NA |
|  | rs385076 | GIANT | G | G/A | 0.75 | 0.25 | 0.025 | 0.0091 | 0.0067 | + | NA |
| *IL18* | rs71478720 | DIAGRAM | C | C/G | NA | NA | 0.05827 | 0.01926 | 0.0029 | + | NA |
|  | rs71478720 | NHLBI GRASP Catalog (version 2) | NA | NA | NA | NA | NA | NA | 2.58E-06 | NA | NA |
|  | rs71478720 | DIAGRAM | C | C/G | NA | NA | 0.05827 | 0.01926 | 0.0031 | + | NA |
|  | rs71478720 | DIAGRAM | A | A/G | NA | NA | 0.05827 | 0.01926 | 0.0031 | + | NA |
|  | rs71478720 | IBDGC | A | A/G | NA | NA | NA | NA | 0.00976 | NA | NA |
|  | rs71478720 | DIAGRAM | C | C/G | NA | NA | 0.06766 | 0.01908 | 0.00068 | + | NA |
|  | rs71478720 | NHLBI GRASP Catalog (version 2) | NA | NA | NA | NA | NA | NA | 2.05E-06 | NA | NA |
|  | rs71478720 | NHLBI GRASP Catalog (version 2) | NA | NA | NA | NA | NA | NA | 2.02E-06 | NA | NA |
|  | rs71478720 | NHGRI-EBI GWAS Catalog | NA | NA | NA | NA | NA | NA | 1.00E-08 | NA | NA |
|  | rs71478720 | NHGRI-EBI GWAS Catalog | NA | NA | NA | 0.24 | NA | NA | 1.00E-08 | NA | NA |
|  | rs71478720 | NHLBI GRASP Catalog (version 2) | NA | NA | NA | NA | NA | NA | 1.02E-08 | NA | NA |
|  | rs71478720 | DIAGRAM | G | G/T | NA | NA | 0.04879 | 0.01945 | 0.008 | + | NA |
|  | rs71478720 | Hom G | G | G/T | NA | NA | NA | NA | 0.000675 | NA | NA |
|  | rs71478720 | DIAGRAM | C | C/T | NA | NA | 0.05827 | 0.01926 | 0.0038 | + | NA |
|  | rs71478720 | MAGIC | C | C/T | NA | 0.193 | -0.057 | 0.022 | 0.009054 | - | NA |
|  | rs71478720 | MAGIC | A | A/C | NA | 0.217 | -0.057 | 0.021 | 0.008273 | - | NA |
|  | rs71478720 | DIAGRAM | T | T/C | NA | NA | 0.05827 | 0.01926 | 0.0047 | + | NA |
|  | rs71478720 | MAGIC | T | T/C | NA | 0.217 | -0.057 | 0.021 | 0.008481 | - | NA |
|  | rs71478720 | NHLBI GRASP Catalog (version 2) | NA | NA | NA | NA | NA | NA | 1.94E-05 | NA | NA |
|  | rs71478720 | NHLBI GRASP Catalog (version 2) | NA | NA | NA | NA | NA | NA | 1.22E-06 | NA | NA |
|  | rs71478720 | NHLBI GRASP Catalog (version 2) | NA | NA | NA | NA | NA | NA | 2.01E-11 | NA | NA |
|  | rs71478720 | NHLBI GRASP Catalog (version 2) | NA | NA | NA | NA | NA | NA | 2.11E-06 | NA | NA |
|  | rs71478720 | NHLBI GRASP Catalog (version 2) | NA | NA | NA | NA | NA | NA | 0.009054 | NA | NA |
|  | rs71478720 | NHLBI GRASP Catalog (version 2) | NA | NA | NA | NA | NA | NA | 1.26E-06 | NA | NA |
|  | rs71478720 | NHLBI GRASP Catalog (version 2) | NA | NA | NA | NA | NA | NA | 1.50E-06 | NA | NA |
|  | rs71478720 | NHLBI GRASP Catalog (version 2) | NA | NA | NA | NA | NA | NA | 0.008273 | NA | NA |
|  | rs71478720 | NHLBI GRASP Catalog (version 2) | NA | NA | NA | NA | NA | NA | 1.49E-06 | NA | NA |
|  | rs71478720 | NHLBI GRASP Catalog (version 2) | NA | NA | NA | NA | NA | NA | 0.008481 | NA | NA |
|  | rs71478720 | DIAGRAM | G | G/A | NA | NA | 0.05827 | 0.01926 | 0.0012 | + | NA |
|  | rs71478720 | GIANT | G | G/A | 0.7583 | 0.2417 | -0.018 | 0.0067 | 0.008 | - | NA |
|  | rs71478720 | Hom G | G | G/A | NA | NA | NA | NA | 0.003461 | NA | NA |
|  | rs71478720 | IBDGC | G | G/A | 0.729 | 0.271 | 0.0655 | 0.0191 | 0.000611 | + | 0.6014 |
|  | rs71478720 | IBDGC | G | G/A | NA | NA | NA | NA | 0.00211 | NA | NA |
|  | rs71478720 | IBDGC | G | G/A | 0.729 | 0.271 | 0.0765 | 0.024 | 0.001412 | + | 0.2544 |
|  | rs71478720 | MAGIC | G | G/A | NA | 0.259 | -0.053 | 0.021 | 0.009675 | - | NA |
|  | rs71478720 | NHLBI GRASP Catalog (version 2) | NA | NA | NA | NA | NA | NA | 3.98E-07 | NA | NA |
|  | rs71478720 | NHLBI GRASP Catalog (version 2) | NA | NA | NA | NA | NA | NA | 0.009675 | NA | NA |
|  | rs71478720 | NHLBI GRASP Catalog (version 2) | NA | NA | NA | NA | NA | NA | 4.33E-09 | NA | NA |
| *OCLN* | Index SNP or proxies not available on Phenoscanner | | | | |  |  |  |  |  |  |

Part 4

| Locus | SNP | N | N Cases | N Controls | N Studies | Unit |
| --- | --- | --- | --- | --- | --- | --- |
| *NLRC4* | rs385076 | 34652 | 12882 | 21770 | 15 | log(OR) |
|  | rs385076 | 20672 | 6945 | 13727 | 8 | log(OR) |
|  | rs385076 | 180866 | 0 | 180866 | 3 | SD |
|  | rs385076 | 180866 | 0 | 180866 | 3 | SD |
|  | rs385076 | 181443 | 0 | 181443 | 62 | years |
|  | rs385076 | 34652 | 12882 | 21770 | 15 | log(OR) |
|  | rs385076 | 158602 | 92857 | 65745 | 51 | log(OR) |
|  | rs385076 | 34652 | 12882 | 21770 | 15 | log(OR) |
|  | rs385076 | 157877 | 92320 | 65556 | 51 | log(OR) |
|  | rs385076 | 158300 | 92715 | 65584 | 51 | log(OR) |
|  | rs385076 | 158597 | 92854 | 65743 | 51 | log(OR) |
|  | rs385076 | 158558 | 92844 | 65714 | 51 | log(OR) |
| *IL18* | rs71478720 | 110452 | 26488 | 83964 | 4 | log(OR) |
|  | rs71478720 | 1958 | NA | NA | NA | NA |
|  | rs71478720 | 110452 | 26488 | 83964 | 4 | log(OR) |
|  | rs71478720 | 110452 | 26488 | 83964 | 4 | log(OR) |
|  | rs71478720 | 20672 | 6945 | 13727 | 8 | log(OR) |
|  | rs71478720 | 110452 | 26488 | 83964 | 4 | log(OR) |
|  | rs71478720 | 1958 | NA | NA | NA | NA |
|  | rs71478720 | 1958 | NA | NA | NA | NA |
|  | rs71478720 | NA | NA | NA | NA | NA |
|  | rs71478720 | NA | NA | NA | NA | NA |
|  | rs71478720 | 1958 | NA | NA | NA | NA |
|  | rs71478720 | 110452 | 26488 | 83964 | 4 | log(OR) |
|  | rs71478720 | 3094 | 1311 | 1783 | 3 | log(OR) |
|  | rs71478720 | 110452 | 26488 | 83964 | 4 | log(OR) |
|  | rs71478720 | 15234 | 0 | 15234 | 9 | mmol/l |
|  | rs71478720 | 15234 | 0 | 15234 | 9 | mmol/l |
|  | rs71478720 | 110452 | 26488 | 83964 | 4 | log(OR) |
|  | rs71478720 | 15234 | 0 | 15234 | 9 | mmol/l |
|  | rs71478720 | 173 | NA | NA | NA | NA |
|  | rs71478720 | 1958 | NA | NA | NA | NA |
|  | rs71478720 | 1200 | NA | NA | NA | NA |
|  | rs71478720 | 1958 | NA | NA | NA | NA |
|  | rs71478720 | 45854 | NA | NA | NA | NA |
|  | rs71478720 | 1958 | NA | NA | NA | NA |
|  | rs71478720 | 1958 | NA | NA | NA | NA |
|  | rs71478720 | 45854 | NA | NA | NA | NA |
|  | rs71478720 | 1958 | NA | NA | NA | NA |
|  | rs71478720 | 45854 | NA | NA | NA | NA |
|  | rs71478720 | 110452 | 26488 | 83964 | 4 | log(OR) |
|  | rs71478720 | 60584 | 0 | 60584 | 46 | Z-score |
|  | rs71478720 | 3094 | 1311 | 1783 | 3 | log(OR) |
|  | rs71478720 | 34652 | 12882 | 21770 | 15 | log(OR) |
|  | rs71478720 | 20672 | 6945 | 13727 | 8 | log(OR) |
|  | rs71478720 | 27432 | 6968 | 20464 | 8 | log(OR) |
|  | rs71478720 | 15234 | 0 | 15234 | 9 | mmol/l |
|  | rs71478720 | 1958 | NA | NA | NA | NA |
|  | rs71478720 | 45854 | NA | NA | NA | NA |
|  | rs71478720 | 1200 | NA | NA | NA | NA |
| *OCLN* | Index SNP or proxies not available on Phenoscanner | | | | |  |
